# Effect of surface finishing on the colour stability and translucency of dental ceramics

**DOI:** 10.1186/s12903-018-0508-4

**Published:** 2018-03-13

**Authors:** Işıl Sarıkaya, Kaan Yerliyurt, Yeliz Hayran

**Affiliations:** Department of Prosthodontics, Gaziosmanpasa University Faculty of Dentistry, 60100 Tokat, Turkey

**Keywords:** Ceramic, Colour stability, Surface finishing, Translucency

## Abstract

**Background:**

The purpose of this study was to investigate the effects of staining solutions and surface finishing on the colour stability and translucency of hybrid ceramic (HC) and resin nanoceramic (RNC) materials.

**Methods:**

Twenty four groups consisting of 10 specimens (240 specimens in total) were created out of HC and RNC, including six groups to be stored in distilled water served as the controls groups. The Vita Enamic technical set, Shofu polishers, medium and fine rubber wheels and Sof-Lex polishing discs were used as polishing instruments. Cola, tea, and coffee were used as staining solutions. The colour differences (∆E*) and translucency parameter (TP) were evaluated by a spectrophotometer. Data were analysed by a One-way Analysis of Variance (ANOVA) and Mann-Whitney *U* test.

**Results:**

There was a statistically significant difference between the ∆E* values of the HC specimens in the coffee groups and the ∆E* values of the other HC groups (*p* < 0.05). The ∆E* values of the RNC specimens in the coffee and tea groups were not different from the specimens in the cola groups (*p* > 0.05). The TP values of the polished groups were higher than the Sof-Lex groups and the Shofu groups on both HC and RNC materials (*p* < 0.05).

**Conclusions:**

Increased ∆E* values were observed in HC specimens stored in a coffee solution compared to the specimens stored in a tea or cola solution. Both of the RNC specimens stored in coffee and tea had higher ∆E* values than the RNC specimens stored in the cola. The TP values of both HC and RNC specimens stored in the coffee solution decreased.

## Background

Efforts to strengthen dental ceramics by modifying their microstructures have continued to increase in the last twenty years. Adding a crystalline structure to the glassy matrix of feldspathic porcelain reinforces both the optical and mechanical properties of the ceramic. Alumina- and zirconia-based systems are opaque, whereas leucite-reinforced systems are more translucent [1]. Zirconia-framework materials have low translucency properties because of zirconia’s opaque white colour. Also, feldspathic ceramics are used as a layer that mimics natural tooth colour. Enamic (Vita Zahnfabrik, Bad Sackingen, Germany), one of the polymer-infiltrated feldspathic ceramic materials, consists of 86% ceramic (by weight) [2]. Besides having the properties of both ceramic and composite materials, polymer-infiltrated ceramic-network (PICN) materials are considered to have mechanical and aesthetic properties similar to natural teeth [3]. Lava ultimate (Lava Ultimate; 3M Espe) is a strong, wear-resistant, and highly aesthetic milling block that provides an alternative to ceramic blocks for computer-aided design/computer-aided manufacturing (CAD/CAM) indirect restorations [4].

Smoother restoration surfaces create biologically ideal surfaces by minimising the effect of plaque accumulation and discolouration. If a restoration has a sufficiently smooth surface, it can prevent the formation of biofilm layers and extrinsic stains. When glaze surfaces deform during contour adjustments as incisal/occlusal or facial surfaces, clinical adjustments of the ceramic restorations are generally prone to create aesthetic problems for the patients.

Certainly, colour stability is an important clinical factor in aesthetic dental restorations [5, 6]. Also, the survival rate and aesthetic appearance of ceramic restorations depends on colour stability and translucency. The translucency of dental materials are defined as the translucency parameter (TP) [7]. TP describes the colour difference between a black and a white background. The Commision Internationale de I’Eclairage (CIE) recommends calculating colour difference based on the CIE L*a*b* colour parameters [8]. Color differences (∆E*) in CAD/CAM ceramics are affected by the translucency and the background color [9]. There is also only a limited knowledge on the effect of surface roughness on the color of ceramic after it has been subjected to a staining agent in the literature [10–14].

However, every single material has to be evaluated individually in regard to their mechanical and aesthetic properties. Limited data are available in the literature on the colour stability and translucency of newly introduced CAD/CAM milling blocks. Thus, the purpose of this study was to determine the effects of staining solutions and surface finishing on the colour stability and translucency of hybrid materials. The null hypothesis of this study was that both staining solutions and surface finishing are not correlated with the stainability and translucency of hybrid ceramics (HC) and resin nanoceramics (RNC).

## Methods

### Sampling

Two of the most popular CAD/CAM ceramics were chosen in this study. The selected HC and RNC materials are listed in Table 1. Using a precision saw machine, 1.2 mm–thick disk specimens with 14 mm diameters were prepared with high-translucence CAD/CAM blocks by slicing them with a water-cooled diamond disk (Micracut 201, Bursa, Turkey) at low speeds (150 rpm). The shades of the Enamic (Vita Enamic, Vita Zahnfabrik, Germany) specimens were characterised with a specially developed, white Vita Enamic Stains Kit (Vita Zahnfabrik, Germany) following the manufacturer’s instructions. The 2M2 colour for Enamic specimens was chosen according to the Vita 3D-Master shade guide (Vita Zahnfabrik, Germany) which corresponds with the Lava Ultimate (Lava Ultimate, 3M Espe, USA) specimens’ A2 colour according to the Vitapan Classical shade guide (Vita Zahnfabrik, Germany).

**Table 1:** Materials tested

Material	Enamic	Lava ultimate
Code	HC	RNC
Composition	Hybrid ceramic	Resin nanoceramic
Filler Type	Silica and alumina	Zirconia and silica nanoparticles and nanoclusters
Particle %weight	86	80
Lot no.	51040	33140A2-HT
Manufacturer	Vita Zahnfabrik	3M ESPE
Translucency/shade	HT/2M2	HT/A2

As listed in Table 2, 24 groups consisting of 10 specimens (240 specimens in total) were created out of HC and RNC , including six groups to be stored in distilled water served as the controls groups (E1a,E2a, E3a, L1a, L2a, L3a) . All of the specimens’ surfaces were roughened on both sides with 600-, 800- and 1200-grit silicon carbide abrasive papers (English Abrasives, London, UK) under running water at 100 cycles per minute. This standardised polishing procedure eliminated external irregular scratches of the cut specimens and equally rounded the chipped corners [9]. The specimens in the E1b, E1c and E1d groups were then polished with the Vita Enamic technical polishing set (Vita Zahnfabrik, Bad Sackingen, Germany) following the manufacturer’s instructions for the. Specimens in groups L1b, L1c and L1d were polished with a low-speed hand piece at 10,000 rpm with medium and fine rubber wheels (Dedeco Red and Green Rubber Wheels, Dedeco, NY, USA). The specimens in the E2b, E2c, E2d, L2b , L2c and L2d groups were polished with 12.7 mm–diameter Sof-Lex polishing discs (3M Espe, MN, USA) mounted on a hand piece set at a speed of 10,000 rpm for coarse and medium discs and 30,000 rpm for fine and superfine discs according to the manufacturers’ recommendations. The specimens were polished in succession with a low-speed hand piece at 10,000 rpm with an abrasive stone(Dura Green Stones; Shofu Inc, Kyoto, Japan), a coarse silicon carbide polisher (Ceramaster Coarse; Shofu Inc), and a silicon carbide polisher (Ceramaster; Shofu Inc) in the groups E3b, E3c, E3d, L3b, L3c and L3d. All polishing instruments were applied on both sides of the specimens with a low-speed rotating hand piece (Kavo Ewl 4990; KaVo Dental Gmbh, Germany) by the same investigator.

**Table 2:** Materials, surface finishing and groups

Materials surface finishing and groups				
HC (E)	polishing+distilled	polishing+cola	polishing+tea	polishing+coffee
	water E1a	E1b	E1c	E1d
	sof-lex+distilled	sof-lex+cola	sof-lex+tea	sof-lex+coffee
	water E2a	E2b	E2c	E2d
	shofu+distilled	shofu+cola	shofu+tea	shofu+coffee
	water E3a	E3b	E3c	E3d
RNC (L)	polishing+distilled	polishing+cola	polishing+tea	polishing+coffee
	water L1a	L1b	L1c	L1d
	sof-lex+distilled	sof-lex+cola	sof-lex+tea	sof-lex+coffee
	water L2a	L2b	L2c	L2d
	shofu+distilled	shofu+cola	shofu+tea	shofu+coffee
	water L3a	L3b	L3c	L3d

### Colour measurements

Before colour measurements were taken, all of the specimens were cleaned in deionised water for ten minutes in an ultrasonic cleaner (Pro-Sonic 600; Sultan Healthcare, NJ, USA) and then dried with compressed air. The thickness of the specimens were confirmed with a digital calliper (Absolute Digimatic, Mitutoyo, Japan). The baseline colour measurements were performed with a clinical spectrophotometer (Vita Easy Shade Advance, Vita Zahnfabrik, Germany) under standard illuminant D65 using CIE L*a*b* and were recorded before treatment with the staining solution. All measurements were performed on white, black and neutral grey surfaces. After repeating the colour measurements three times for each of the specimens, the mean values of L*, a* and b* were calculated. In the CIE L*a*b* system color differences (∆E*) formula, L* represent lightness, a* represents the chromaticity coordinate for red-green and b* represents the chromaticity coordinate for yellow-blue for color differences (∆E*) formula [8].

Translucency was determined by identifying the value of the TP. Measurements were performed with the spectrophotometer under D65 illumination over white and black backgrounds. Measurements were repeated three times, and the mean CIE L*a*b* values were recorded for both backgrounds. The TP was obtained by calculating the difference in colour between the specimen over a white and black background with the following formula [15, 16]:

$$ TP={\left({\left({L^{\ast}}_B-{L^{\ast}}_W\right)}^2+{\left({a^{\ast}}_B-{a^{\ast}}_W\right)}^2+{\left({b^{\ast}}_B-{b^{\ast}}_W\right)}^2\right)}^{1/2} $$

In this formula, *B* signifies the colour coordinates over a black background and *W* signifies the colour coordinates over a white background. The greater the TP value, the higher the translucency of the material. A TP value of 100 indicates the specimen is transparent and a TP value of 0 indicates that the material is opaque.

After the baseline colour measurements were made, the specimens were stored in four different solutions. The tea solution was prepared with 200 ml of boiling water poured over a 2 g tea bag (Lipton, Unilever, Turkey) according to the manufacturer’s recommendations. The coffee solution was made with 3.6 g of coffee (Nescafe Classic; Nestle, Bursa, Turkey) grounds and 300 ml of boiled water according to the manufacturers’ recommendations. The solution was filtered through a filter paper after 10 min of storing. A 48 h storage period was selected with reference to previous studies [17, 18]. The solution was stirred every 8 ± 1 hours. A Coca-Cola solution (The Coca-Cola Company, İstanbul, Turkey) was prepared with a can of 330 ml of Coca-Cola. Finally, distilled water served as the control group’s solution. All specimens were stored in 100 ml solutions at 37°C for 48 h. Then, the specimens were rinsed with distilled water for 5 min and blotted dry with tissue paper (Selpak; Eczacıbaşı, Istanbul, Turkey) before colour measurements.

The second set of colour measurements were made using the spectrophotometer with the same method as the first. The determination of the colour variation, ∆E*, between the two colour measurements was made in the CIE L*a*b* system using the following equation:

$$ \Delta {E}^{\ast }={\left({\left({L_1}^{\ast }-{L_2}^{\ast}\right)}^2+{\left({a_1}^{\ast }-{a_2}^{\ast}\right)}^2+{\left({b_1}^{\ast }-{b_2}^{\ast}\right)}^2\right)}^{1/2}. $$

To evaluate whether the colour differences in translucent ceramics were acceptable, this study used an average threshold of ∆E* = 2.7 [19].

### Statistical analysis

Data are shown as mean and standard deviation. Three way Anova was used for main effects and interaction effects accoring to three factors. Bonferroni correction was used for multiple comparisons. With 80% power, 5% margin of error and effect size of 0.242, there are totally 240 samples obtain as 240/24 = 10 samples for each group. Analysis were conducted using commercial software (IBM SPSS Statistics 20, IBM Inc., Somers NY, USA). Values of *p < 0.05* were considered significant.

## Results

### Colour differences

**Fig. 1:**
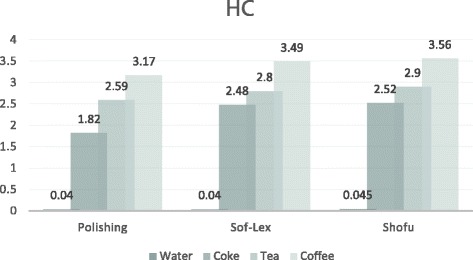
Comparison of the *∆E* values of different groups of HC material

**Fig. 2:**
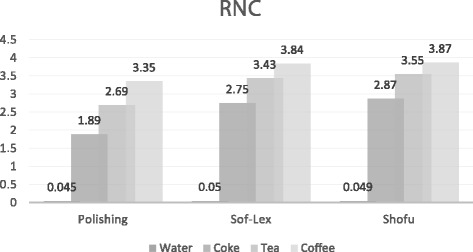
Comparison of the *∆E* values of different groups of RNC material

The one-way ANOVA test results and the means and standard deviations of ∆E* values are shown in Table 3. No significant differences in ∆E* values were detected between groups E1a, E2a, E3a, L1a, L2a and L3a, which were the six control groups (*p* > 0.05). Also, no significant differences in ∆E* values were observed within the polished HC groups (E1a, E1b, E1c, E1d), the Sof-Lex HC groups (E2a, E2b, E2c, E2d) or the Shofu HC groups (E3a, E3b, E3c, E3d) (*p* > 0.05 for all 3). The ∆E* values were significantly different among the polished RNC groups (L1a, L1b, L1c, L1d), the Sof-Lex RNC groups (L2a, L2b, L2c, L2d), and the Shofu RNC groups (L3a, L3b, L3c, L3d) (*p* < 0.05 for all 3). The ∆E* values of HC specimens in the polished group (E1d), Sof-Lex group (E2d) and Shofu group (E3d) that were stored in the coffee solution had statistically significant differences from the HC groups (*p* < 0.05). The ∆E* values of the RNC specimens in the polishing group (L1c, L1d), Sof-Lex group (L2c, L2d), and Shofu group (L3c, L3d) that were stored in the tea and coffee solutions were significantly different from the specimens in the groups (L1b, L2b, L3b) stored in the cola solution (*p* < 0.05). A comparison of the ∆E* values of the different groups of the HC and RNC materials are shown in Figs. 1 and 2.

**Table 3:** Mean and SD of *ΔE* values and differences between groups

		Distilled water	Cola	Tea	Coffee
HC	polishing	0.04±0.005 A,x,p	1.82±0.31 B,x,p	2.59±0.35 C,x,p	3.17±0.32 D,x,p
	(n=10)				
	sof-lex	0.04±0.005 A,x,p	2.48±0.45 B,x,q	2.80±0.35 B,x,p	3.49±0.59 C,x,p
	(n=10)				
	shofu	0.045±0.003 A,x,p	2.52±0.17 B,x,q	2.90±0.47 B,x,p	3.56±0.36 C,x,p
	(n=10)				
RNC	polishing	0.045±0.005 A,x,p	1.89±0.24 B,x,q	2.69±0.59 C,x,q	3.35±0.40 C,x,p
	(n=10)				
	sof-lex	0.05±0.009 A,y,p	2.75±0.28 B,x,p	3.43±0.27 C,y,p	3.84±0.85 C,x,pq
	(n=10)				
	shofu	0.049±0.012 A,x,p	2.81±0.35 B,y,p	3.55±0.36 C,y,p	3.87±0.46 C,x,q
	(n=10)				

*Different laters indicate statistically significant difference between groups (*p*<0.05)

^a^A,B,C,D intra-group comparisons

^b^x,y between-group comparisons column

^c^p,q column-group comparisons

### Translucency Parameter

**Fig. 3:**
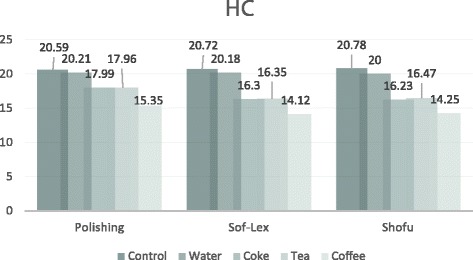
Comparison of the TP values of different groups of HC material

**Fig. 4:**
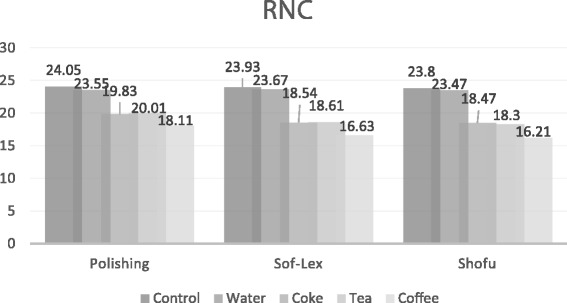
Comparison of the TP values of different groups of RNC material

The one-way ANOVA results and the means and standard deviations of the TP values are shown in Table 4. No significant differences in the TP values were detected between the specimens in the groups E1a, E2a, E3a, L1a, L2a and L3a which were stored in distilled water as the control groups (*p* > 0.05). However, these groups showed the highest TP values out of all HC and RNC groups. The TP values were significantly higher with the HC and RNC polished groups than the HC and RNC Sof-Lex groups and Shofu groups (*p* < 0.05). Also, the TP values of the HC and RNC specimens stored in the coffee solution were the lowest, which was significantly different than the specimens stored in cola, and tea (*p* < 0.05). The TP values of both HC and RNC specimens with polishing, Sof-Lex finishing and Shofu finishing that were stored in tea and cola were not significantly different from one another (*p* > 0.05). The RNC specimens also showed higher TP values than the HC specimens in all groups, and these values were statistically different from each other (*p* < 0.05). A comparison of the TP values of the different groups of HC and RNC materials are shown in Figs. 3 and 4.

**Table 4:** Mean and SD of TP values and differences between groups

		Control	Distilled water	Cola	Tea	Coffee
HC	polishing	20.59±0.58	20.21±1.94	17.99±0.94	17.96±0.97	15.35±0.97
	(n=10)	A,p,x	A,p,x	B,p,x	B,p,x	C,p,x
	sof-lex	20.72±1.76	20.18±1.13	16.30±0.92	16.35±1.07	14.12±0.74
	(n=10)	A,p,x	A,p,x	B,q,x	B,q,x	C,q,x
	shofu	20.78±1.25	20.00±0.78	16.23±1.67	16.47±0.59	14.25±1.76
	(n=10)	A,p,x	A,p,x	B,q,x	B,q,x	C,q,x
RNC	polishing	24.05±0.43	23.55±1.62	19.83±1.18	20.01±1.40	18.11±0.75
	(n=10)	A,p,y	A,p,y	B,p,y	B,p,y	C,p,y
	sof-lex	23.93±1.17	23.67±1.19	18.54±0.84	18.61±0.80	16.63±1.22
	(n=10)	A,p,y	A,p,y	B,q,y	B,q,y	C,q,y
	shofu	23.80±1.26	23.47±1.70	18.47±1.12	18.30±3.11	16.21±0.87
	(n=10)	A,p,y	A,p,y	B,q,y	B,q,y	C,q,y

*Different laters indicate statistically significant difference between groups (*p*<0.05)

^a^A,B,C intra-group comparisons

^b^p,q between-group comparisons column

^c^x,y column-group comparisons

## Discussion

Based on the results of this study, the null hypothesis that both staining solutions and surface finishing are not correlated with the stainability and translucency of HC and RNC materials was rejected. In this study, an increase in ∆E* values was observed in the cola, tea and coffee groups for both HC and RNC specimens but not for specimens stored in distilled water as the control group. These groups also exceeded the TP values of control groups. The TP values of these groups ranging from highest to lowest were those stored in the cola, tea and coffee solutions for both HC and RNC materials.

Johnston *et al*. [20] reported an acceptability threshold of ∆E* = 3.7 as a limit, and this limit has been referenced for many years. Many of the different studies on dental ceramics consider a range of ∆E* = 2 to 4 as the acceptability threshold. [5, 21, 22]. Also, colour perception is related to many factors, such as an individual’s colour perception, the material’s surface texture, illumination conditions and instrumental differences in colour matching [23]. In this study, the colour difference in translucent ceramics was accepted as ∆E* = 2.7, which has been reported as an average threshold value in previous studies [19]. There has been no clear agreement about the accepted ∆E* limit until the present day [23]. In the present study, the ∆E* values of HC and RNC materials stored in the coffee solution for all groups were higher than the accepted threshold ∆E* value. Also, this situation is valid for ∆E* values of HC and RNC materials stored in tea solutions for all groups finished with Sof-Lex and Shofu.

Several reports have investigated different polishing techniques for ceramic restorations to create smooth surfaces, such as glazing, and support the use of polishing as an alternative to glazing [9, 24–26]. Another, significant factor in colour stability is the type of surface treatment. [9, 19, 27, 28]. Coffee has been the most frequently used staining solution in colour studies followed by tea and cola [9, 17, 19, 25–29].

In a previous study that investigated the effects of tea, coffee and cola on the colour of resins and ceramics, it was reported that colour change of porcelain was not noticeable (ΔE*=1.2 to 1.4) [30]. In another study, a glazed ceramic material’s colour change after immersion in coffee was found to be less than a composite resin’s colour change [31].

In a recent study, Acar *et al.* [32] evaluated optical properties of nanocomposite resins and ceramics at various thicknesses due to thermocycling in coffee. They reported that thermocycling in coffee caused a clinically unacceptable colour change for Lava Ultimate and Filtek Supreme Plus, and the colour changes of Enamic were perceptible but clinically acceptable. Also, they concluded that when colour stainability with coffee is considered, Enamic may be an alternative to lithium disilicate ceramic restorations fabricated with minimally invasive techniques. Unlike the present study, a spectroradiometer was used to measure the spectral radiance of the specimens. A limitation of the present study was that thermal cycling was not used for coffee staining. However, like the present study, Lava Ultimate was showed more discolouration than the Enamic in Acar *et al*.’s study.

Similar to the present study, in a recent study Awad *et al*. [33] reported that Lava Ultimate was more translucent than Enamic with two different specimen thicknesses (1mm and 2mm). CAD/CAM ceramics at all 3 surface conditions, which were polished surfaces and surfaces grinded with 1200 grit and 500 grit SiC grinding sheets. Also, they concluded that Enamic achieved the lowest TP values because of the high amount of Al_2_O_3_ (approximately 23 wt%). Moreover, the material composition strongly influenced translucency. Lava Ultimate is an RNC containing 80 wt% silica and zirconia nanoparticles and nanoclusters bound in the resin matrix. The ceramic particles are made up of three different ceramic fillers that reinforce a highly cross-linked polymeric matrix, which is comprised of 20 nm silica and 4–11 nm zirconia particles. In a previous study, Lava Frame was found to be the most translucent material among the different zirconia materials by the direct transmission method and light flow [23, 30, 34].

Vita Enamic was reported to be the best choice for anterior and posterior restorations that closely matched neutral tooth colour in the literature [35]. In Enamic, the ceramic-network material is infiltrated with urethane dimethacrylates (UDMA) and triethylene glycol dimethacrylate (TEGDMA) mixture [36]. Because TEGDMA has higher water absorption, staining agents more easily penetrate the resin matrix. Therefore, the stainability of Enamic may be due to the TEGDMA content [37].

HC and RNC blocks are fabricated based on two levels of precrystallisation treatment. HT material contains a small number of large crystals in the precrystallised matrix, whereas LT material contains a large number of smaller crystals. Furthermore, opacity increases with the thickness for all ceramic materials. A TP range of 5.5 to 7.1 for highly translucent composites, 3.8 to 5.4 for moderately translucent composites, and 2.0 to 3.7 for more opaque composites [38]. As a limitation of this study, both HC and RNC specimens were prepared in a single thickness. Further studies are required to determine the TP of HC and RNC materials with different thicknesses.

## Conclusions

Within the limitations of this in vitro study, the following conclusions were drawn: Increased ∆E* values were observed in HC specimens stored in a coffee solution compared to the specimens stored in a tea or cola solution. Both of the RNC specimens stored in coffee and tea had higher ∆E* values than the RNC specimens stored in the cola. The TP values of both HC and RNC specimens stored in the coffee solution decreased. RNC specimens showed higher TP values than HC specimens for all groups. Coffee has caused the most stained surfaces among tea and cola on HC and RNC. According to the results of the present study, Sof-Lex finishing and Shofu finishing of Enamic surfaces may be an alternative to finishing with Vita Enamic technical polishing set for handling less stainability of the material’s.

## Abbreviations

%: Percent
g: Gram
h: Hour
min: Minute
ml: Milliliter
mm: Millimeter
rpm: Revolutions per minute
3D: 3-Dimentional
wt: Weight
